# New biomarkers of Kawasaki disease identified by gingival crevicular fluid proteomics

**DOI:** 10.3389/fmolb.2025.1597412

**Published:** 2025-05-12

**Authors:** Xue Fan, Ying Li, Yuehao Xu, Jianqing Lin, Xin Guo, Jinwen Liao, Mingguo Xu

**Affiliations:** ^1^ Clinical Research Institute, Shanghai General Hospital, Shanghai Jiao Tong University School of Medicine, Shanghai, China; ^2^ Department of Pediatrics, Shenzhen People’s Hospital, Shenzhen, China; ^3^ Department of Pediatrics, The Third People’s Hospital of Longgang, Clinical Institute of Shantou University Medical College, Shenzhen, China; ^4^ Department of Pediatrics, Longgang District Maternal and Child Healthcare Hospital of Shenzhen City, Shenzhen, China

**Keywords:** Kawasaki disease, coronary artery lesions, bioinformatics analysis, gingival crevicular fluid, proteomics

## Abstract

**Introduction:**

Kawasaki disease (KD) is an acute systemic vasculitis that primarily affects coronary arteries, and delayed diagnosis increases the risk of cardiovascular complications. Biomarkers are essential for improving diagnostic accuracy, especially in atypical cases. Gingival crevicular fluid (GCF), derived from periodontal tissues, contains serum components and inflammatory mediators, and has emerged as a valuable biofluid for systemic disease diagnosis. Previous studies suggest GCF protein profiles reflect immune status and metabolic disorders, such as type 2 diabetes. Given the immune-related nature of KD, GCF protein composition may also be altered, yet no studies have systematically explored GCF biomarkers in KD. This study uses DIA and MRM-MS proteomics to identify potential GCF biomarkers for KD diagnosis.

**Methods:**

Twenty-seven patients with KD were enrolled in this study, and 18 healthy volunteers were recruited as the control group. GCF samples were collected from the KD patients, who formed the experimental group, before they received intravenous immunoglobulin treatment. Data-independent acquisition (DIA) quantitative proteomics mass spectrometry was performed on the GCF samples to analyze the protein expression profiles in both groups. DEPs were identified and subjected to functional enrichment analysis using KEGG and GO. Protein–protein interaction (PPI) analysis was conducted for all detected DEPs. Finally, multiple reaction monitoring mass spectrometry (MRM-MS) was used to validate the selected DEPs.

**Results:**

A total of 197 DEPs were identified in GCF between the KD group and the normal control group, with 174 upregulated and 23 downregulated proteins. Functional enrichment analysis revealed that cellular and metabolic processes were the most significantly altered biological processes, while binding and catalytic activity were the most affected molecular functions. Pathway analysis further highlighted the NOD-like receptor signaling pathway, protein processing in the endoplasmic reticulum, and the influenza pathway as the most significantly enriched pathways. In the PPI network, EIF2AK2, B2M, and GBP1 were identified as key hub proteins, suggesting their potential regulatory roles in KD pathophysiology. Finally, MRM-MS confirmed the expression patterns of 12 DEPs (IFIT3, UB2L6, HP, A1AT, HSP90AA1, HNRPC, HSP90AB1, SAA1, MX1, B2M, FKBP4, and TRAP1), thereby demonstrating high consistency with the DIA results and further validating the DEPs’ potential as biomarkers for KD.

**Conclusion:**

Our findings suggest that 12 proteins in GCF could serve as potential biomarkers for the early diagnosis of KD. Additionally, the molecular analysis revealed a close association between KD and gingival inflammation, offering new insights into KD’s pathophysiology and potential directions for improved diagnosis and treatment.

## 1 Introduction

Kawasaki disease (KD) is an acute, self-limiting systemic vasculitis syndrome that primarily affects the arteries, particularly the coronary arteries. Delayed diagnosis increases the risk of arterial damage, making timely and accurate diagnosis and treatment essential for reducing cardiac complications and preserving long-term health and quality of life (Hu et al., 2019).

Biomarkers play a vital role in precision diagnosis and may help identify atypical KD cases, thereby reducing the incidence of cardiovascular complications. Gingival crevicular fluid (GCF), a biofluid derived from periodontal tissues, contains a mixture of serum components and locally generated inflammatory mediators. GCF is considered rich in multiple immune factors and can be used as a diagnostic test for periodontal disease and certain systemic disorders (Chaparro et al., 2013). Besides plasma and urine, GCF is one of the major human biofluids focused on for clinical diagnosis use in recent years (Chaparro et al., 2016). It is an exudate from periodontal tissues and is composed of serum and locally generated materials that can be used as diagnostic tests for periodontal disease and other systemic disorders, such as Sjogren syndrome and type 2 diabetes (Mohamed et al., 2015). GCF proteins have been reported to change significantly in type 2 diabetes compared to healthy control, diabetic, and prediabetic subjects. This indicates that GCF can be used as a source of biomarkers for diabetes mellitus (Barros et al., 2016).

KD is a disease closely related to immune status, which may lead to alterations in GCF protein composition. However, to date, no systematic assessment of potential GCF biomarkers for KD diagnosis has been conducted. KD typically induces small-to medium-sized vasculitis, which may also affect gingival tissue and lead to changes in GCF composition. To investigate these differences between KD patients and healthy volunteers, mass spectrometry-based proteomics has been applied. Data-independent acquisition (DIA) efficiently detects protein molecules with very low abundance in complex samples, enabling high-throughput, accurate, and reproducible data analysis (Song et al., 2017). Multiple reaction monitoring mass spectrometry (MRM-MS) allows for protein identification without the need for specific antibodies, offering the advantage of precise simultaneous quantification of at least 100 protein targets per sample (Xu et al., 2020).

In this study, we employ DIA-based proteomics to screen for differentially expressed proteins (DEPs) and validate the findings using MRM-MS. To the best of our knowledge, this is the first study to characterize GCF protein profiles in KD patients, and it highlights the potential of GCF biomarkers for KD diagnosis.

## 2 Materials and methods

### 2.1 Subjects

A total of 28 KD patients diagnosed at Shenzhen Children’s Hospital between October and December 2019 were included, along with 18 healthy volunteers recruited as the control group. The study was approved by the Ethics Committee of Shenzhen Children’s Hospital (approval no. 202003802), and written informed consent was obtained from the guardians of all participants. No significant differences were observed in the average age or male-to-female ratio between the two groups.

### 2.2 GCF collection

The patients’ GCF was collected when KD was diagnosed and before immunoglobulin treatment was performed. The GCF of the healthy children was collected during physical examinations in the children’s health department of our hospital. All participants (KD patients and healthy controls) were screened for periodontal disease and oral hygiene status. Individuals with active periodontal disease or poor oral hygiene were excluded. Using a disposable oral examination kit, the tooth surface was dried with a sterile cotton ball, and a moisture-absorbing paper tip (Beijing Dayading) was inserted into the gingival crevicular area. The paper tip was taken out after 30°s, put into an EP tube. Samples were immediately stored at −80°C to preserve protein integrity, and freeze-thaw cycles were avoided. GCF collection was performed by a single trained operator to minimize inter-operator variability. However, if blood was found on the paper tip, it was discarded.

### 2.3 GCF protein extraction

A 5 mm steel bead was added to each sample, and the protein was extracted with lysis buffer containing 1 mM PMSF and 2 mM EDTA (final concentration). After 5 min, 10 mM DTT (final concentration) was added to the samples. After centrifugation at 4°C and 25,000 g, the supernatant was discarded. To reduce disulfide bonds in the proteins of the supernatant, 10 mM DTT (final concentration) was added and incubated at 56°C for 1 h. Subsequently, 55 mM IAM (final concentration) was added to block the cysteines and incubated for 1 h in a darkroom. The supernatant was mixed well with a 4× volume of chilled acetone for 2 h at −20°C to precipitate proteins until the supernatant was colorless (repetition of addition if necessary). An appropriate amount of lysis buffer three was added to the processed sample for precipitation, followed by ultrasonication to dissolve the precipitated proteins. After centrifugation, the supernatant was taken for quantification.

### 2.4 Protein digestion

Total protein (100 μg) was taken out of each sample solution and digested at 37°C for 16 h with trypsin gold (Promega, Madison, WI, United States) at a ratio of 30:1 protein to trypsin. After trypsin digestion, the peptides were dried by vacuum centrifugation and reconstituted in 0.5 M TEAB.

### 2.5 High pH reverse-phase separation

A total of 10 μg was taken from all samples to form a 200 μg mixture, which was diluted with 2 mL mobile phase A buffer (5% ACN pH 9.8) and injected into a Shimadzu LC-20AB HPLC system coupled with a Gemini high pH C18 column (5 um, 4.6 × 250 mm). The sample was subjected to the column and then eluted at a flow rate of 1 mL/min by gradient (5% mobile phase B [95% CAN, pH 9.8] for 10 min, 5%–35% mobile phase B for 40 min, 35%–95% mobile phase B for 1 min). Flow phase B lasted 3 min, and 5% mobile phase B was equilibrated for 10 min. The elution peak was monitored at a wavelength of 214 nm, and the components were collected every minute. These components were combined into 10 fractions, which were then freeze dried.

### 2.6 DIA analysis by nano-LC-MS/MS

The peptides were separated by liquid phase chromatography, ionized using a nanoESI source, and passed to a Q-Exactive HF X tandem mass spectrometer (Thermo Fisher Scientific, San Jose, CA) in DIA detection mode. The main parameter settings were as follows: ion source voltage was set to 1.9 kV, MS1 scanning range was 400∼1250 m/z, resolution was set to 120,000, maximum ion implantation time (MIT) was 50 m, and 400–1250 m/z was equally divided into 50 continuous windows for fragmentation and signal acquisition. The ion fragmentation mode was HCD, the maximum ion implantation time was selected as the automatic mode, the fragment ions were detected in Orbitrap, the resolution was set to 30,000, the fragmentation energy was distributed fragmentation (22.5, 25, and 27.5), and AGC was set to 1E6.

### 2.7 MRM-MS verification

The samples were digested as described and spiked with 50 fMol of β-galactosidase for data normalization. MRM analyses were performed on a QTRAP 6500 mass spectrometer (SCIEX, Framingham, MA, United States) equipped with an LC-20AD nanoHPLC system (Shimadzu, Kyoto, Japan). The mobile phase consisted of solvent A (0.1% aqueous formic acid) and solvent B (98% acetonitrile with 0.1% formic acid). Peptides were separated on a C18 column (0.075 × 150 mm column, 3.6 μm) at 300 nL/min and eluted with a gradient of 5%–30% solvent B for 38 min, 30%–80% solvent B for 4 min, and maintenance at 80% for 8 min. For the QTRAP 6500 mass spectrometer, a spray voltage of 2400 V, nebulizer gas of 23 psi, and a dwell time of 10 ms were used. Multiple MRM transitions were monitored using unit resolution in both the Q1 and Q3 quadrupoles to maximize specificity.

### 2.8 Statistical analysis

DEPs in the KD, compared to normal control samples, were identified with an abundance ratio of two or more or less than 0.5, and a p-value of less than 0.05 was considered significant. GO and KEGG enrichment analyses were performed using Phyper, a function of R. The significant levels of terms and pathways were corrected by a Q-value with a rigorous threshold (Q-value <0.05). Moreover, by using the String database (https://string-db.org/), a protein–protein interaction (PPI) network of DEPs was obtained.

The results are presented as mean ± standard deviation (SD). To confirm a significant difference between specific groups, group comparisons were performed using the Student’s t-test. In all cases, a p-value of less than 0.05 was considered a statistically significant difference.

## 3 Results

### 3.1 DEPs in GCF

**FIGURE 1:**
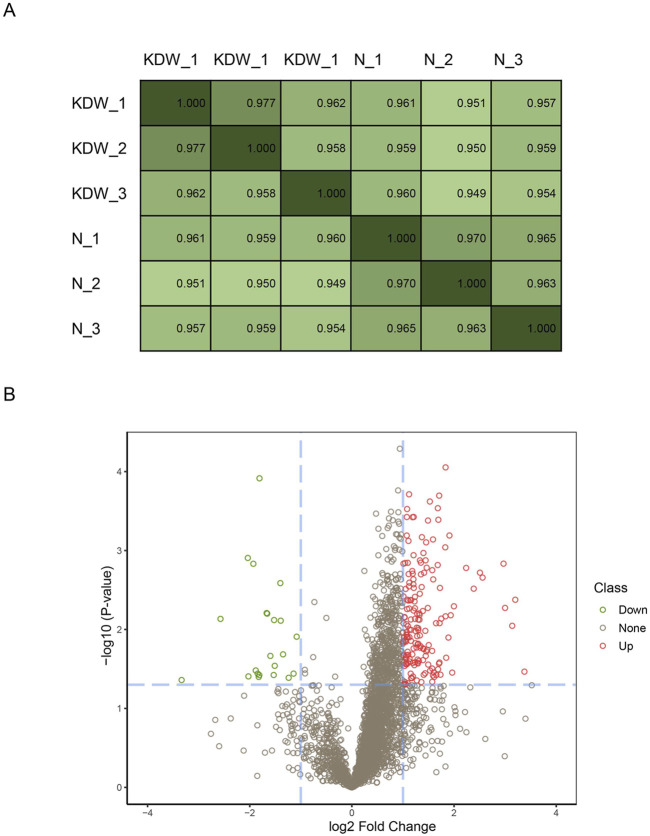
Repeats steadity and differentially expressed proteins. **(A)** Pearson coeffiency test of biological repeats. **(B)** Volcano plot of differential proteins. Fold change (absolute value of fold change) >2 was the standard screening differential protein, based on *p* < 0.05. Differential proteins are significantly upregulated in the red part and significantly downregulated in the green part of the figure.

A total of 3,353 proteins were detected using DIA. We performed Pearson correlation coefficient tests to evaluate the biological repeats. It turned out that all three biological repeats correlated well, with the least being 0.958 (Figure 1A). After basic analysis, we discovered 197 significant DEPs in the KD group compared to the healthy controls (Figure 1B), of which 174 DEPs were upregulated and 23 were downregulated.

### 3.2 GO and KEGG enrichment analysis of DEPs

**FIGURE 2:**
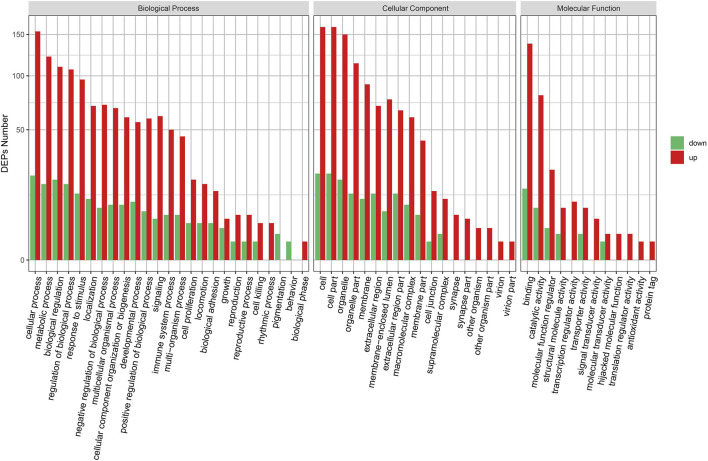
GO enrichment of DEPs.GO enrichment plot of differential proteins. The horizontal axis is the number of differential proteins, and the vertical axis is the GO-enriched entries.

We performed GO analyses of all the DEPs identified in the KD group. We found that the DEPs were mainly involved in the upregulation of the biological process, the cellular component, and the molecular function (Figure 2). The cellular and metabolic processes, binding, and catalytic activity were the most altered biological processes and molecular functions. Furthermore, cell killing, biological phase, supramolecular complex, synapse, molecular transducer activity, hijacked molecular function, translation regulator activity, antioxidant activity, and protein tag were the only upregulation GO terms.

**FIGURE 3:**
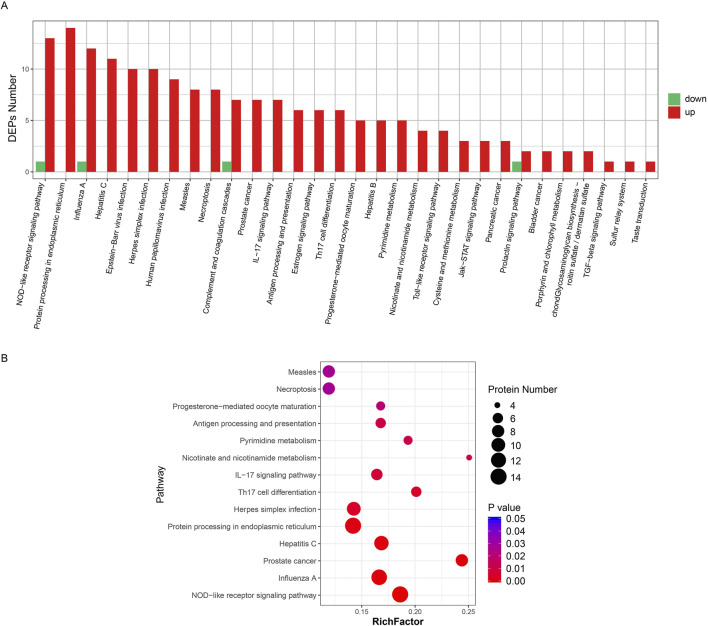
Pathway enrichment of all DEPs. **(A)** Cluster plot of differential protein expression. The horizontal axis is the pathway name and the vertical axis is the number of DEPs found in the KD group. **(B)** Pathway enrichment of differential proteins. The horizontal axis is the group name and the vertical axis is the protein ID of the differential protein. The legend on the right shows the metrics for the differential proteins (converted from p-values to fold change, not added in parentheses). In the figure, the horizontal axis is the number of differential proteins and the vertical axis is the pathway enrichment entries.

There were 30 enriched pathways of DEPs in the KD group compared with the normal group (Figure 3A). Similar to GO enrichment, 26 pathways contained only upregulation DEPs. In addition, 14 significantly enriched pathways are shown in Figure 3B, including the NOD-like receptor signaling pathway, protein processing in the endoplasmic reticulum pathway, and the influenza pathway.

### 3.3 Protein–protein interaction network analysis of DEPs

**FIGURE 4:**
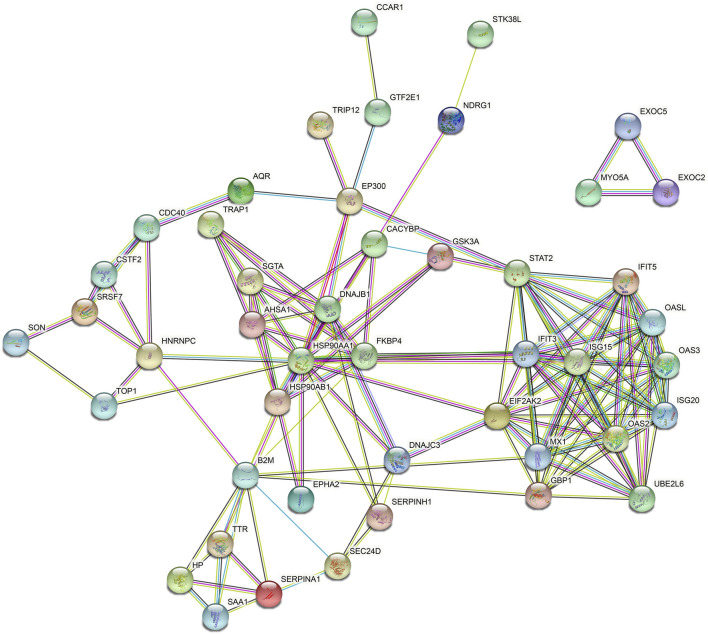
Differential protein interaction network. Red circles indicate protein upregulation and blue circles indicate protein downregulation. The degree of denseness of the junction line represents the degree of tightness of the relationship between this protein and other differential proteins.

The DEPs were subjected to interaction analysis by comparing them with the STRING database, and network interactions were plotted taking the top 100 interaction relationships with confidence (Figure 4). It could be seen that PPI in the KD DEPs mostly consisted of two networks. The bigger network was a complex interaction composed of 42 proteins, and the smaller one was an independent three-protein triangle network. EIF2AK2, B2M, and GBP1 are kernel proteins that are able to interact with more proteins.

### 3.4 Validation of protein biomarkers in KD

**FIGURE 5:**
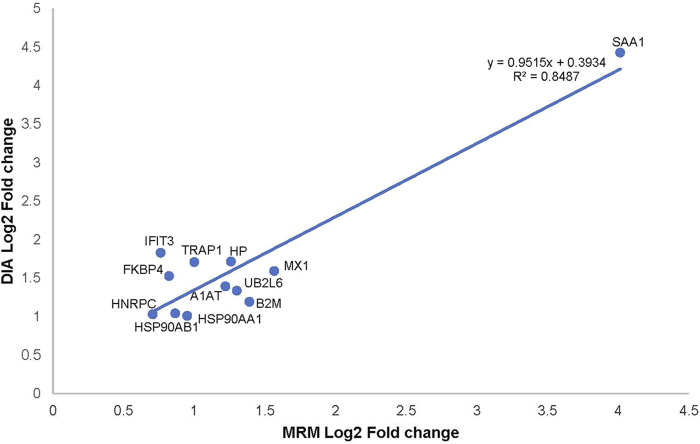
Correlation plot between the expression of alternative protein markers detected by MRM and DIA.

We chose 12 significant DEPs from the PPI network for validation using MRM-MS, which showed that the results of MRM and DIA tended to be consistent (Figure 5). Combined with a p-value of less than 0.05, it can be considered that the results detected by the two detection methods were positively correlated. An *R* ^2^ of 0.9387 (i.e., >0.85) meant that the linear regression fitting was good, indicating a correlation. Table 1 shows that the validated DEPs were selected using a p-value of less than 0.05 and a fold change greater than 1.5, all of which were upregulated.

**TABLE 1:** Protein markers in GCF of KD verified by MRM.

Protein	Fold change	*P* Value	adj.pvalue
IFIT3	1.695	0.023	0.025
UB2L6	2.464	0.000	0.000
HPT	2.393	0.001	0.001
A1AT	2.329	0.000	0.000
HS90A	1.932	0.000	0.000
HNRPC	1.630	0.000	0.000
HS90B	1.822	0.000	0.000
SAA1	16.159	0.000	0.000
MX1	2.959	0.000	0.000
B2MG	2.619	0.000	0.000
FKBP4	1.766	0.000	0.000
TRAP1	1.999	0.000	0.000

## 4 Discussion

In this study, we extracted GCF samples from KD patients and healthy volunteers and performed DIA quantitative proteomics identification and MRM validation for DEPs between the two groups. We identified 197 DEPs from 3,353 proteins; of these, 174 were significantly upregulated and 23 were significantly downregulated. Then, 46 potential alternative protein markers were found in the GCF of KD patients using PPI network analysis. Subsequently, we performed quantitative protein detection using MRM-MS to validate 12 DEPs, which was consistent with the results we obtained from DIA detection.

The results of the GO enrichment analysis showed that the DEPs in the GCF of KD patients were mainly enriched in biological processes, including cell proliferation, immune system processes, and cellular processes. The main signaling pathways involved in DEPs were cell growth and death, post-transport catabolism, cell motility, cardiovascular diseases, infectious diseases, bacteria, and immune diseases. Based on previously published data, we found that cell proliferation, migration, death, and immune processes play a key role in the pathogenesis of KD and the occurrence of vascular injury (Xu et al., 2020). The presence of related proteins in GCF samples suggests that GCF can be used as a biomarker resource for KD diagnosis (Kumrah et al., 2020). The GCF could be used as a sample to investigate the pathomechanisms of periodontitis (Tsuchida et al., 2018). The present research revealed that the bacterial infection pathway was also enriched in GCF in patients with KD, which indicates that pathogen infection may be involved in the pathogenesis of KD and periodontitis. To screen the key protein markers in GCF for KD, we selected 12 DEPs (SAA1, FKBP4, IFIT3, UB2L6, HPT, A1AT, HS90A, HNRPC, HS90B, MX1, B2MG, and TRAP1) detected by DIA to verify using MRM-MS. Interestingly, these results were all consistent with the data of DIA.

SAA1 is one of the major proteins of amyloid A, which plays an important role in lipid metabolism, bacterial infection, arterial inflammation, and tumor (Getz et al., 2016; Sun and Ye, 2016). Chen et al. (2020) reported an association between the genetic locus polymorphisms of SAA1 and coronary artery disease in KD, indicating that SAA1 may be involved in the process of coronary artery injury. Increased levels of SAA1 protein in human periodontal lesion tissues are positively correlated with periodontal inflammation, and SAA1 may induce inflammatory cell infiltration and release inflammatory factors through toll-like receptor two and toll-like receptor 4 (Hirai et al., 2019). The present study showed that the protein expression of SAA1 was significantly increased in the GCF of patients with KD, which suggests that SAA1 may be closely related to the pathogenesis of periodontal tissue and vessel inflammation.

The dynein-associated immunoaffinity FKBP52 (FKBP4) belongs to the immunoaffinity protein family and plays an important role in the immunoregulatory processes, protein folding, and trafficking activities associated with heat shock protein 90 (HSP90) (Xiong et al., 2020). FKBP4 interacts with HSP90 to regulate the activity of the steroid receptor axis (Lagadari et al., 2016). Data have shown that FKBP4-deficient mice can develop specialized phenotypes associated with androgen, progesterone, and glucocorticoid insensitivity (Guy et al., 2015). HSP90 is a chaperone protein that regulates protein maturation and is involved in the regulation of atherosclerotic lesions through various pathways, such as lipid metabolism disorders (Zheng et al., 2019), vascular smooth muscle cell proliferation and migration (Fu et al., 2019), glucocorticoid receptor axis (Profumo et al., 2018), and oxidative stress (Zhang et al., 2017). Xu et al. (2020)’s study confirmed that the traditional Chinese medicine berberine can protect against oxidative stress damage to coronary endothelial cells in KD by inhibiting HSP90B. HSP90 has also been demonstrated to be involved in the immune response to periodontal inflammation due to *Porphyromonas gingivalis* infection (Sweier et al., 2009). The results of this study suggest that FKBP4 and HSP90A/B expression are synergistically increased in KD, revealing that FKBP4 and HSP90A/B may be involved in the regulatory process of gingival injury in patients with KD (Shelburne et al., 2007).

Interferon-induced protein repeats with tetratricopeptide 3 (IFIT3) is an interferon-inducible protein with antiviral and proinflammatory effects. IFIT3 can be used as a biomarker of the macrophage-polarizing proinflammatory phenotype (M1) and is upregulated in the arterial tissue of atherosclerotic mice (Huang et al., 2018). MX1 has antipathogenic and proinflammatory functions, which have been reported to be associated with the depletion of vascular endothelial progenitor cells and endothelial dysfunction (Lee et al., 2007). Studies have shown that MX1 is involved in the regulation of pathogen defense mechanisms in gingival tissues (Mahanonda et al., 2012). IFIT3 and MX1 are important mediators of inflammation and vascular diseases and may be involved in the pathogenesis of KD and periodontal diseases.

The E2 ubiquitin-conjugating enzyme UB2L6 (UB2L6) belongs to the ubiquitin–proteasome system and is used to transport ubiquitin and promote substrate proteins to complete ubiquitin labeling, which is associated with the regulation of both apoptosis and the cell cycle (Pintado et al., 2012). The increased expression level of UB2L6 in the present experiment suggested an increased level of ubiquitination in the gingival tissue of KD, but the molecular regulatory mechanism involved was still unclear. Tumor necrosis factor receptor–associated protein 1 (TRAP1, or HSP75) is a major member of the HSP90 family and can prevent cardiomyocyte injury induced by hypoxia by maintaining mitochondrial activity (Li et al., 2020). Increased TRAP1 expression may indicate a protective function in gingival tissue in patients with KD.

Studies have confirmed that the expression of α1-antitrypsin (A1AT) is increased in the plasma of patients with KD, which can inhibit the neutrophil elastase activity associated with coronary artery damage induced by KD (Yu et al., 2009). Published data have confirmed that A1AT is also associated with periodontal inflammation and may identify the severity of periodonatal status (Song et al., 2020). Data have also shown that A1AT has antiviral and protective effects in lung diseases (de Loyola et al., 2020), while the role of A1AT in the cardiovascular system has not yet been clearly reported (Curjuric et al., 2018). In the present study, the expression of A1AT was upregulated, as mentioned earlier. Further experiments should be performed to investigate the role of A1AT in the pathogenesis of KD and periodontal diseases.

The sample size of this study is relatively small, which may limit the robustness of the findings. Future studies with larger cohorts, including stratified analyses based on clinical stages of KD, are warranted to validate the identified biomarkers. In addition, future studies should include patients with periodontal disease as controls to further evaluate the specificity of the identified biomarkers for KD.

## 5 Conclusion

We found that there were 174 DEPs in GCF between the control group and the KD group, most of which were associated with cell growth and death, post-transport catabolism, cell motility, cardiovascular disease, infectious diseases, bacteria, and immune diseases. Among the 174 DEPs, 12 proteins were validated using MRM-MS, including IFIT3, UB2L6, HPT, A1AT, HS90A, HNRPC, HS90B, SAA1, MX1, B2MG, FKBP4, and TRAP1, which could be used as potential biomarkers for KD diagnosis. The clinical utilizability of the present results should be investigated via further basic research.

## Data Availability

The data are not publicly available due to institutional and ethical constraints related to pediatric patient privacy. Requests to access the datasets should be directed to the corresponding author.

## References

[B1] Barros S. Williams R. Offenbacher S. Morelli T. (2016). Gingival crevicular fluid as a source of biomarkers for periodontitis. Periodontol. 2000 70 (1), 53–64. 10.1111/prd.12107 26662482 PMC4911175

[B2] Chaparro A. Gaedechens D. Ramírez V. Zuñiga E. Kusanovic J. Inostroza C. (2016). Placental biomarkers and angiogenic factors in oral fluids of patients with preeclampsia. Prenat. Diagn. 36 (5), 476–482. 10.1002/pd.4811 26988336

[B3] Chaparro A. Sanz A. Quintero A. Inostroza C. Ramirez V. Carrion F. (2013). Increased inflammatory biomarkers in early pregnancy is associated with the development of pre-eclampsia in patients with periodontitis: a case control study. J. Periodontal Res. 48 (3), 302–307. 10.1111/jre.12008 23035752

[B4] Chen Y. Wang C. Ji Q. Zhang J. Tan C. Wang S. (2020). Association of rs4638289 and rs7131332 polymorphisms of the serum amyloid A1 gene with Kawasaki disease. Chin. J. Contemp. Pediatr. 22 (6), 614–619. 10.7499/j.issn.1008-8830.1912093 PMC7390225 32571461

[B5] Curjuric I. Imboden M. Bettschart R. Caviezel S. Dratva J. Pons M. (2018). Alpha-1 antitrypsin deficiency: from the lung to the heart? Atherosclerosis 270, 166–172. 10.1016/j.atherosclerosis.2018.01.042 29432934

[B6] de Loyola M. Dos Reis T. de Oliveira G. da Fonseca Palmeira J. Argañaraz G. Argañaraz E. (2020). Alpha-1-antitrypsin: a possible host protective factor against Covid-19. Rev. Med. Virol. 31, e2157. 10.1002/rmv.2157 32844538 PMC7461031

[B7] Fu W. Chen M. Ou L. Li T. Chang X. Huang R. (2019). Xiaoyaosan prevents atherosclerotic vulnerable plaque formation through heat shock protein/glucocorticoid receptor axis-mediated mechanism. Am. J. Transl. Res. 11 (9), 5531–5545. 31632527 PMC6789251

[B8] Getz G. Krishack P. Reardon C. (2016). Serum amyloid A and atherosclerosis. Curr. Opin. Lipidol. 27 (5), 531–535. 10.1097/MOL.0000000000000331 27579547

[B9] Guy N. Garcia Y. Cox M. (2015). Therapeutic targeting of the FKBP52 co-chaperone in steroid hormone receptor-regulated physiology and disease. Curr. Mol. Pharmacol. 9 (2), 109–125. 10.2174/1874467208666150519114115 25986565

[B10] Hirai K. Furusho H. Kawashima N. Xu S. de Beer M. Battaglino R. (2019). Serum amyloid A contributes to chronic apical periodontitis via TLR2 and TLR4. J. Dent. Res. 98 (1), 117–125. 10.1177/0022034518796456 30189157 PMC6304714

[B11] Hu H. Du H. Cui J. Feng D. Du Z. (2019). New biomarkers of Kawasaki disease identified by urine proteomic analysis. FEBS Open Bio 9 (2), 265–275. 10.1002/2211-5463.12563 PMC6356163 30761252

[B12] Huang C. Lewis C. Borg N. Canals M. Diep H. Drummond G. (2018). Proteomic identification of interferon-induced proteins with tetratricopeptide repeats as markers of M1 macrophage polarization. J. Proteome Res. 17 (4), 1485–1499. 10.1021/acs.jproteome.7b00828 29508616

[B13] Kumrah R. Vignesh P. Rawat A. Singh S. (2020). Immunogenetics of Kawasaki disease. Clin. Rev. Allergy Immunol. 59 (1), 122–139. 10.1007/s12016-020-08783-9 32200494

[B14] Lagadari M. De Leo S. Camisay M. Galigniana M. Erlejman A. (2016). Regulation of NF-κB signalling cascade by immunophilins. Curr. Mol. Pharmacol. 9 (2), 99–108. 10.2174/1874467208666150519113833 25986566

[B15] Lee P. Li Y. Richards H. Chan F. Zhuang H. Narain S. (2007). Type I interferon as a novel risk factor for endothelial progenitor cell depletion and endothelial dysfunction in systemic lupus erythematosus. Arthritis Rheum. 56 (11), 3759–3769. 10.1002/art.23035 17968925

[B16] Li X. Li Y. Shi Z. Guo X. (2020). New insights into molecular chaperone TRAP1 as a feasible target for future cancer treatments. Life Sci. 254, 117737. 10.1016/j.lfs.2020.117737 32376268

[B17] Mahanonda R. Sa-Ard-Iam N. Rerkyen P. Thitithanyanont A. Subbalekha K. Pichyangkul S. (2012). MxA expression induced by α-defensin in healthy human periodontal tissue. Eur. J. Immunol. 42 (4), 946–956. 10.1002/eji.201141657 22531919

[B18] Mohamed H. Idris S. Ahmed M. Åstrøm A. Mustafa K. Ibrahim S. (2015). Influence of type 2 diabetes on local production of inflammatory molecules in adults with and without chronic periodontitis: a cross-sectional study. BMC Oral Health 15, 86. 10.1186/s12903-015-0073-z 26211001 PMC4515322

[B19] Pintado C. Gavilán M. Gavilán E. García-Cuervo L. Gutiérrez A. Vitorica J. (2012). Lipopolysaccharide-induced neuroinflammation leads to the accumulation of ubiquitinated proteins and increases susceptibility to neurodegeneration induced by proteasome inhibition in rat hippocampus. J. Neuroinflammation 9, 87. 10.1186/1742-2094-9-87 22559833 PMC3462674

[B20] Profumo E. Buttari B. Tinaburri L. D’Arcangelo D. Sorice M. Capozzi A. (2018). Oxidative stress induces HSP90 upregulation on the surface of primary human endothelial cells: role of the antioxidant 7,8-dihydroxy-4-methylcoumarin in preventing HSP90 exposure to the immune system. Oxid. Med. Cell. Longev. 2018, 2373167. 10.1155/2018/2373167 29849874 PMC5914108

[B21] Shelburne C. Coopamah M. Sweier D. An F. Lopatin D. (2007). HtpG, the Porphyromonas gingivalis HSP-90 homologue, induces the chemokine CXCL8 in human monocytic and microvascular vein endothelial cells. Cell Microbiol. 9 (6), 1611–1619. 10.1111/j.1462-5822.2007.00897.x 17346315

[B22] Song L. Gou W. Wang J. Wei H. Lee J. Strange C. (2020). Overexpression of alpha-1 antitrypsin in mesenchymal stromal cells improves their intrinsic biological properties and therapeutic effects in nonobese diabetic mice. Stem Cells Transl. Med. 10, 320–331. 10.1002/sctm.20-0122 32945622 PMC7848369

[B23] Song Y. Zhong L. Zhou J. Lu M. Xing T. Ma L. (2017). Data-independent acquisition-based quantitative proteomic analysis reveals potential biomarkers of kidney cancer. Proteomics Clin. Appl. 11. 10.1002/prca.201700066 28975715

[B24] Sun L. Ye R. (2016). Serum amyloid A1: structure, function and gene polymorphism. Gene 583 (1), 48–57. 10.1016/j.gene.2016.02.044 26945629 PMC5683722

[B25] Sweier D. Shelburne P. Giannobile W. Kinney J. Lopatin D. Shelburne C. (2009). Immunoglobulin G (IgG) class, but not IgA or IgM, antibodies to peptides of the Porphyromonas gingivalis chaperone HtpG predict health in subjects with periodontitis by a fluorescence enzyme-linked immunosorbent assay. Clin. Vaccine Immunol. 16 (12), 1766–1773. 10.1128/CVI.00272-09 19793900 PMC2786377

[B26] Tsuchida S. Satoh M. Takiwaki M. Nomura F. (2018). Current status of proteomic technologies for discovering and identifying gingival crevicular fluid biomarkers for periodontal disease. Int. J. Mol. Sci. 20 (1), 86. 10.3390/ijms20010086 30587811 PMC6337088

[B27] Xiong H. Chen Z. Zheng W. Sun J. Fu Q. Teng R. (2020). FKBP4 is a malignant indicator in luminal A subtype of breast cancer. J. Cancer 11 (7), 1727–1736. 10.7150/jca.40982 32194784 PMC7052866

[B28] Xu M. Qi Q. Men L. Wang S. Li M. Xiao M. (2020). Berberine protects Kawasaki disease-induced human coronary artery endothelial cells dysfunction by inhibiting of oxidative and endoplasmic reticulum stress. Vasc. Pharmacol. 127, 106660. 10.1016/j.vph.2020.106660 32070767

[B29] Yu H. Kuo H. Sheen J. Wang L. Lin I. Wang C. (2009). A unique plasma proteomic profiling with imbalanced fibrinogen cascade in patients with Kawasaki disease. Pediatr. Allergy Immunol. 20 (7), 699–707. 10.1111/j.1399-3038.2008.00844.x 19170925

[B30] Zhang X. Shi H. Wang Y. Hu J. Sun Z. Xu S. (2017). Down-regulation of hsa-miR-148b inhibits vascular smooth muscle cells proliferation and migration by directly targeting HSP90 in atherosclerosis. Am. J. Transl. Res. 9 (2), 629–637. 28337290 PMC5340697

[B31] Zheng Z. Zhang X. Liu X. Jin X. Dai L. Cheng H. (2019). Inhibition of HSP90β improves lipid disorders by promoting mature SREBPs degradation via the ubiquitin-proteasome system. Theranostics 9 (20), 5769–5783. 10.7150/thno.36505 31534518 PMC6735373

